# Daily Financial Thoughts and Indices of Mental and Physical Health: The Importance of Socioeconomic Status

**DOI:** 10.1093/geroni/igab046.1516

**Published:** 2021-12-17

**Authors:** Jennifer Piazza, Jonathan Rush, Eric Cerino, Jacqueline Mogle, Robert Stawski, Susan Charles, David Almeida

**Affiliations:** 1 California State University, Fullerton, California State University, Fullerton, California, United States; 2 University of Victoria, Victoria, British Columbia, Canada; 3 Northern Arizona University, Flagstaff, Arizona, United States; 4 Penn State University, University Park, Pennsylvania, United States; 5 Oregon State University, Corvallis, Oregon, United States; 6 University of California, Irvine, Irvine, California, United States; 7 Pennsylvania State University, University Park, Pennsylvania, United States

## Abstract

The current study examined the associations between daily financial thoughts, socioeconomic status (SES), and indices of emotional (positive and negative affect (PA/NA)) and physical health (physical symptoms and cortisol). Participants (N = 782) from the National Study of Daily Experiences, a subsample of the Midlife in the United States Refresher survey, completed daily diary interviews and provided saliva samples, from which cortisol was assayed. Participants who, on average, reported more daily financial thoughts also reported more NA, less PA, more physical symptoms, and had higher cortisol AUCg (all p’s < .05). These effects were more pronounced among people reporting lower SES. Daily fluctuations in financial thoughts also predicted daily fluctuations in NA, PA, and physical symptoms (all p’s <. 01). Again, these associations were more pronounced among people reporting lower SES. Results indicate that intrusive, daily financial thoughts may be one pathway explaining the link between SES and health outcomes.

